# Existence of Neural Stem Cells in Mouse Spleen

**DOI:** 10.1155/2019/6264072

**Published:** 2019-01-09

**Authors:** Koichi Tomita, Hiroshi Ishikawa

**Affiliations:** ^1^Department of Digestive and Transplantation Surgery, Tokyo Medical University Hachioji Medical Center, 1163 Tatemachi, Hachioji, Tokyo 193-0998, Japan; ^2^Laboratory of Clinical Regenerative Medicine, Department of Neurosurgery, Faculty of Medicine, University of Tsukuba, 1-1-1 Tennodai, Tsukuba, Ibaraki 305-8575, Japan

## Abstract

Pluripotent stem cells are used in regenerative medicine and exist in various internal organs. However, there are a small number of reports of neural cells or neural stem cells existing in the spleen. In this study, we sought to identify possible neural stem cells in the mouse spleen. The spleens of ICR mice were removed and small specimens were incubated in Dulbecco's modified Eagle's medium with Nutrient Mixture F-12 containing either 10% fetal bovine serum (FBS), 20% FBS, 10% neonate bovine serum, or 10% fetal calf serum. Neural cell medium was also used. The cultured cells were investigated for expression of the neural cell markers neuron-specific enolase (NSE) and neurofilament 150 kDa (NF-150) by immunocytochemistry. Mouse spleens were also examined by immunohistochemistry for NSE, NF-150, NF-200, peripherin, and glial fibrillary acidic protein. Cells morphologically resembling neural cells were obtained and were positive for neural cell markers. Some of the cells generated sphere-like formations, which may have been neurospheres. Cell proliferation was best in medium containing 10% FBS. Cells positive for neural markers were observed in the subcapsular and perivascular regions of the spleen. The cells were round and present in much lower numbers than in cell culture. These cells are suspected neural stem cells and would be expected to differentiate into neural cells in cell culture. This report suggests the existence of neural stem cells in the mouse spleen.

## 1. Introduction

Neural stem cells or neural progenitor cells have received much attention in regenerative medicine, because the nervous system has only limited ability to regenerate and several neural diseases remain incurable. Neural stem cells have been reported to exist in various internal organs and tissues, such as the brain [1–3]. The spleen is not a vital organ in humans or rodents and is resectable if necessary. This would make the spleen a useful source of neural stem cells. However, although the organ does contain a population of naturally occurring stem cells [4, 5], there are small number of reports about the existence of neural stem cells in the spleen. In this study, we have cultured neural cells from mouse spleens and demonstrated the existence of neural cell marker-positive cells in the mouse spleen.

## 2. Material and Methods

### 2.1. Animals

Six-week-old ICR mice were used in this study. The mice were housed in the animal facility at 24°C with appropriate humidity. Food and drink were provided ad libitum. Animals were maintained in accordance with the guidelines issued by the Institutional Animal Ethics Committee, and the Institutional Review Board approved this study. The mice were sacrificed without any suffering as mentioned below.

### 2.2. *In Vitro* Cell Culture

The cell culture protocol in this study was modified from previously reported methods [6–9]. The mice were sacrificed by cervical dislocation under deep anaesthesia with intraperitoneal injection of Nembutal, and their spleens were aseptically removed. Each spleen was placed in a 10-cm plastic culture dish (Falcon, USA) with growth medium, minced with a pair of scalpels, and incubated at 37°C in a humidified atmosphere containing 4.7% CO2. Culture was performed under aseptic conditions in a laminar air flow chamber.

The growth medium used was Dulbecco's modified Eagle's medium containing Nutrient Mixture F-12 (DMEM/F12; Cat. 12500; Gibco, Grand Island, NY, USA) supplemented with 0.1% nonessential amino acid solution (Lot 1133557), 0.25 *μ*g/mL fungizone, 50 U/mL penicillin, and 50 *μ*g/mL streptomycin (all from Gibco), and one of the following sera: 10% fetal bovine serum (FBS; Lot 24300113; Moregate, Australia), 20% FBS, 10% neonate bovine serum (NBS; Lot 6202B028; Biocell, California, USA), and 10% fetal calf serum (FCS; Lot PN69; Nakashibetsu, Mitsubishi Kasei Corporation, Tokyo, Japan).

In addition, neural cell medium [10] was used, which consisted of DMEM/F12 (Gibco), 1% N2 supplement (Gibco), 100 *μ*M ascorbic acid (Sigma, St. Louis, MO, USA), 5 ng/mL epidermal growth factor (Sigma), and 5 *μ*g/mL linoleic acid (Sigma).

The growth medium was changed twice weekly, before the medium color changed from red to yellow. The cells were passaged to 80–90% confluence. During culture, the cells were periodically observed with an IMT-2 inverted phase contrast microscope (Olympus, Tokyo, Japan) and imaged.

### 2.3. *In Vitro* Immunocytochemistry

Immunocytochemistry was performed as previously described, with modifications [9], using cells cultured in DMEM/F12 containing 10% FBS. Attached cells were dissociated with 0.25% trypsin (DIFCO, Sparks, MD, USA)/0.02% ethylenediaminetetraacetic acid (Gibco), collected by centrifuge, and disseminated into each well of an eight-chamber Tissue-Tek slide (Nalgene Nunc International, Rochester, NY, USA) at 1.0–1.5×10^3^ cells/cm^2^. Culture was continued with the same growth medium until adherent cells displayed extended cellular processes. At the end of culture, the cells were fixed with 4% paraformaldehyde (PFA) in 0.1 M sodium phosphate buffer (SPB) for 30 minutes at room temperature.

After fixation, the cells were rinsed with phosphate-buffered saline (PBS), incubated with 5% normal goat or donkey serum for 30 minutes at 4°C, and then reacted the primary antibody for 3–5 hours at room temperature. The primary antibodies used were anti-neuron-specific enolase (NSE) rabbit serum (diluted 1:2000; raised in our laboratory and previously validated [11]) and anti-neurofilament 150 kDa (NF-150) rabbit IgG (1:500; B1981; Chemicon International Inc., Temecula, CA, USA).

After several rinses with PBS, the cells were reacted with the secondary antibody, Alexa Fluor 488-labeled goat anti-rabbit IgG (1:500; A11055; Molecular Probes Inc., Leiden, The Netherlands), for 12–20 hours at 4°C in the dark. After several rinses with PBS, the cells were mounted with 0.05 M Tris-HCl buffer (pH 8.0) containing 90% (v/v) nonfluorescent glycerine and 10 mg/mL 1,4-diazabicyclo2.2.2octane. The cells were observed with a confocal laser scanning microscope (LSM 510 META, Carl Zeiss, Germany).

### 2.4. *In Vivo* Immunohistochemistry

Immunohistochemistry was performed as previously described, with modifications [12]. Under deep anaesthesia with 40–50 mg/kg sodium pentobarbital, each mouse received a thoracotomy and was perfused from the left ventricle with a fixative consisting of 4% PFA in 0.1 M SPB. The spleen was removed and further fixed in the same solution for 5 hours. Then, the spleen was hemisected through the longitudinal axis and rinsed with SPB. The specimen was sequentially immersed in 10% and 20% sucrose in PBS. It was then sectioned to 7–12 *μ*m using a sliding microtome equipped with a cold stage, placed on a glass slide, and air dried.

The sections were rinsed with PBS and immunostained with primary antibodies. In addition to NSE and NF-150, anti-neurofilament 200 kDa (NF-200) rabbit IgG (1:500; N4142; Sigma), anti-peripherin goat IgG (1:50; sc-7604; Santa Cruz Biotechnology, Inc., Dallas, TX, USA), and anti-glial fibrillary acidic protein (GFAP) rabbit IgG (1:2000; Z-0334; Dako Cytomation, Denmark) antibodies were also used. Alexa Fluor 488-labeled donkey anti-goat IgG (1:500, A11070, Molecular Probes Inc.) was used as a secondary antibody. The sections were then mounted and observed with confocal laser scanning microscope (same as above).

## 3. Results

### 3.1. *In Vitro* Cell Culture

**Figure 1:**
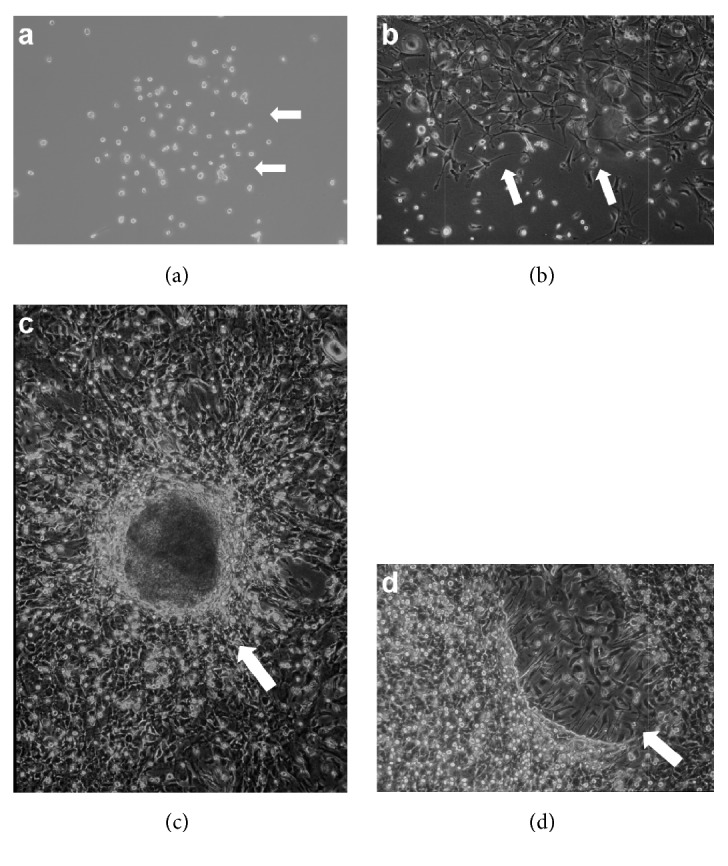
*In vitro* culture of mouse spleen cells in medium containing 10% FBS. The round cells at the beginning of culture (a). Some of them displayed neural cell-like morphology (b). A bright sphere formed (c) and a large number of cells radiated out (d).

*10*%* FBS.* At the beginning of culture, small, round cells attached to the plates (Figure 1(a)). During the first week of the cultivation, a large number of floating blood cells were observed, though these decreased in number and disappeared in a few weeks. The cells rapidly proliferated in most dishes, and became confluent in several weeks. Some adherent cells displayed a neural cell-like appearance approximately one week after the beginning of culture (Figure 1(b)). Bright spheres composed of apolar cells formed after a few weeks of cultivation (Figure 1(c)). A large number of cells radiated out from the spheres into the surroundings (Figure 1(d)).

The proliferation rate of cells was higher in this medium than in the other four growth media, as was the proportion of cells extending processes (data was not shown). The majority of these cells were bipolar. Spherical structures were also recognized in the other media, although they were smaller and fewer in number than in 10% FBS.

**Figure 2:**
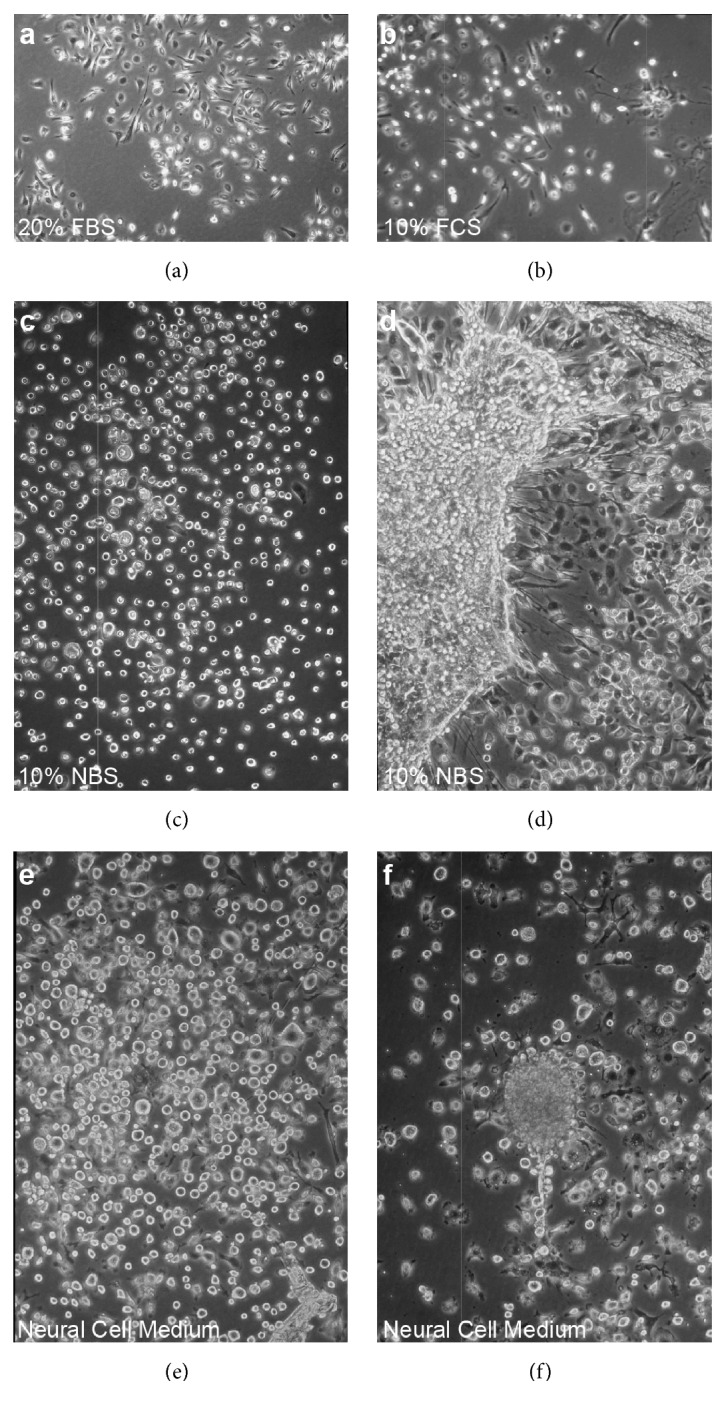
*In vitro* culture of mouse spleen cells in various media (1-4 weeks). Cells were cultured in DMEM/F12 containing 20% FBS (a), 10% FCS (b), or 10% NBS (c, d) or in neural cell medium (e, f). Round to oval shaped cells were dominant in the neural cell medium (e), and some formed spheres (f).

*20*%* FBS.* The appearance and behavior of cells in growth medium containing 20% FBS was similar to that in 10% FBS. However, the spheres were not as prominent in the former (Figure 2(a)).

*10*%* FCS.* In growth medium containing 10% FCS, approximately half of cells were round or oval shaped. Few cells had short processes (Figure 2(b)).

*10*%* NBS.* Most cells in growth medium containing 10% NBS were round, and had no processes (Figure 2(c)). The round cells were scattered throughout the dish and spherical formations were observed (Figure 2(d)). Cells in 10% FCS and 10% NBS proliferated more slowly, requiring over one month to reach confluence.

*Neural Cell Medium.* Round to oval shaped cells were dominant in the neural cell medium, and were scattered throughout the dishes (Figure 2(e)). The cells displayed large variations in size, with large cells 5–7 times bigger than small cells, although even the largest cells were mononuclear. Some spherical formations were observed (Figure 2(f)), as well as a small number of cells with extended processes. The proliferation rate was faster than with 10% FCS and 10% NBS, but not as rapid as with 10% FBS.

### 3.2. *In Vitro* Immunocytochemistry

**Figure 3:**
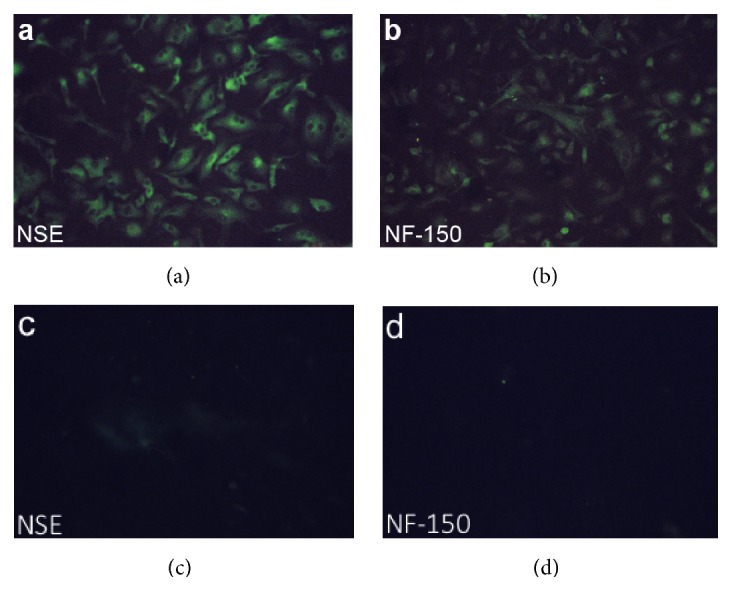
Expression of neural cell markers in cells grown in 10% FBS (2 weeks). Staining for NSE (a) and NF-150 (b) was positive in cells with polars. Negative control of NSE (c) and NF-150 (d) was also shown.

*NSE.* Cells positive for NSE were observed throughout the well (Figure 3(a)). The immunofluorescence was localized to the cytoplasm and the processes, not the nucleus. The processes were stained brightly up to the tip, so that each was clearly indicated.

*NF-150.* Cells positive for NF-150 were also prominent (Figure 3(b)). Immunostaining was mainly localized in the cytoplasm, though the processes were unclear or spottily stained.

### 3.3. *In Vivo* Immunohistochemistry

**Figure 4:**
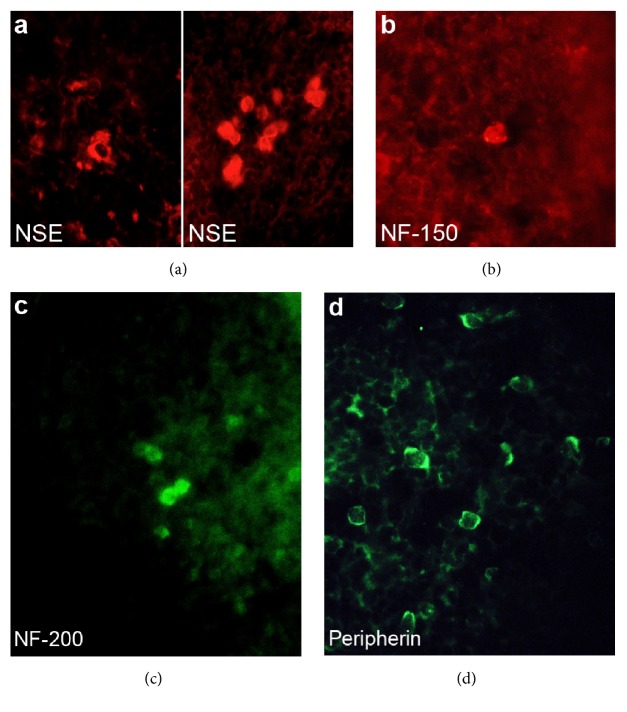
Expression of neural cell markers in the mouse spleen. NSE (a), NF-150 (b), NF-200 (c), and peripherin (d) staining was observed in the perivascular region of the white pulp and the subcapsular region. They were round, with a 10 *μ*m diameter and no recognizable processes.

The immunohistochemical localization of the neural markers was investigated using cryostat section of mouse spleens. A few cells in the pericapsular region and the perivascular region of the white pulp displayed immunoreactivity against NSE (Figure 4(a)), NF-150 (Figure 4(b)), NF-200 (Figure 4(c)), and peripherin (Figure 4(d)). The immunoreactivity for NSE, NF-150, and NF-200 was localized in the cytoplasm of polygonal cells measuring 10 *μ*m in size, and no processes were observed. Cells immunoreactive for GFAP were not detected.

## 4. Discussion

The current study investigated the existence of neural stem cells in the mouse spleen, and the results indicate that the mouse spleen may indeed contain neural stem cells. In cell culture, cells morphologically resembling neural cells were obtained, which were positive for neural cell markers. According to the in vivo study, a small number of cells positive for neural markers were observed in mouse spleens.

The spleen is an organ of the lymphatic system with rich sympathetic innervations [13]. However, a small number of reports have indicated the presence of neural cell perikaryons in the spleen. In addition, the spleen has haematopoietic stem cells [14] and also has potential of expanding stem cells derived from other organs [15–17], though subtractive proteomics of mouse spleen cells indicate no evidence of neural stem cells [18]. This report suggests the existence of mouse spleen neural stem cells.

During cell culture, a number of cells resembling neural cells with extended processes were observed. In addition, some round cells generated sphere-like formations, surrounded by extended processes. Neural stem cells aggregate and form neurospheres during their differentiation into neural cells [3, 19]. This indicates that the aggregated cells in this study may be neurospheres.

On the contrary, cells cultured in neural cell medium were round and did not extend processes comparing to cells cultured with other media. The differences in appearance between neural cell medium and other media may depend on whether they contain serum or not. Serum is known to cause neural cell differentiation [20]. The round cells observed in neural cell medium were probably undifferentiated and immature, as it did not contain serum.

Cells positive for neural cell markers were also observed in the subcapsular and perivascular regions of mouse spleens* in vivo,* although at much lower levels than in cell culture. These cells were apolar and polygonal in shape, and would be expected to proliferate and extend processes during cell culture.

The possibility of splenic stem cells has been investigated in previous studies. There is osteogenic potential in the rat spleen [21]. Another study reported the presence of a cluster of fetal stem cells in the adult spleens of humans and animals, which are CD45^−^ and therefore nonlymphoid cells [4]. The CD45^−^ cell subset, located in the subcapsular region, is proposed to be a remnant of an embryonic stem cell region called the aorta gonad mesoderm (AGM). They expressed Hox11 gene, which are required for development of the nervous system [22, 23]. According to the previous reports, splenic stem cells can promote the development or regeneration of cranial neurons. Hox11 mice without a spleen have cranial nerve problems and NOD mice with defective Hox11 stem cell with transplantation can regenerate, restore portions of nerves in the inner ear and VIII cranial nerve. Also, the Hox11 stem cells of fetal mice that home in the spleen are known to in adult form the mandible, ear, salivary glands, hindbrain, spinal cord and cranial ganglia [24–26]. The immunoreactive subcapsular cells observed in this study might represent these fetal stem cells, though the expression profile of transcription factors including Hox11 was not investigated in this study.

Also, previous study suggests that splenic stem cells express 5 transcription factors of embryonic stem cells [4]. Three of those transcription factor contributes to neuron development (NOTCH3, WNT, and SHH). Another report suggests the brain and cranial nerves were related to splenic stem cells [27]. These may suggest that spleen has neural stem cell.

Further investigation is needed to confirm definitively whether the cells obtained in this study are neural stem cells, including the use of other neural cell markers, and gene expression analysis of the cultured cells.

## 5. Conclusions

In this study, we have demonstrated the existence of neural stem cells in the spleen for the first time. This suggests that the spleen may be a promising source of neural stem cells for use in regenerative medicine, although further experiments will be needed to confirm this.

## Data Availability

The data used to support the findings of this study are available from the corresponding author upon request.
